# Physiological Responses to Chilling Stress and Transcriptome-Assisted Genome-Wide Characterization of the DREB Gene Family in Durian

**DOI:** 10.3390/ijms27188065

**Published:** 2026-09-10

**Authors:** Chaofan Zheng, Jiaying Sheng, Chonghao Zhong, Yikai Wang, Meng Wang, Jiaquan Huang, Hua Tang

**Affiliations:** 1 School of Tropical Agriculture and Forestry, Hainan University, Haikou 570228, China; 13103028307@163.com (C.Z.); 15240571981@163.com (J.S.); 15105485077@163.com (C.Z.); 15969664717@163.com (Y.W.); wm159357zdx@163.com (M.W.); jqhuang@hainanu.edu.cn (J.H.); 2 Institute of Sanya Breeding and Multiplication, Hainan University, Sanya 572025, China; 3 Institute of Baoting, Hainan University, Baoting 572300, China

**Keywords:** *Durio zibethinus*, chilling stress, DREB transcription factor, transcriptome, physiological response, candidate genes

## Abstract

Durian is highly sensitive to chilling temperatures, which restrict its cultivation beyond tropical regions. In this study, one-year-old seedlings of the cultivars ‘Musang King’ and ‘Monthong’ were exposed to 10 °C for 7 days to characterize their phenotypic, physiological, and transcriptional responses. Leaf phenotype, membrane injury, osmotic adjustment, photosynthetic pigment contents, amylase activity, and antioxidant enzyme activities were evaluated, and transcriptome sequencing was performed using samples collected before chilling treatment and after 1 and 5 days of exposure. ‘Monthong’ exhibited earlier and visually more extensive leaf abscission than ‘Musang King’, while the two cultivars showed contrasting temporal patterns of malondialdehyde accumulation and antioxidant enzyme activities. Comparative transcriptome analysis was subsequently used to construct a Musang King-focused discovery set comprising genes prioritized based on progressive induction patterns and higher transcript abundance in ‘Musang King’. Because AP2/ERF transcription factors were represented in this discovery set, the DREB gene family was characterized at the whole-genome level. A total of 81 DzDREB genes were identified and classified into six subgroups. Integration of transcriptomic response patterns, stress-related functional annotations, and promoter cis-regulatory elements supported the prioritization of a ten-gene DzDREB panel for further investigation. Exploratory seasonal field-expression profiling provided additional support for seven candidates, among which *DzDREB36*, *DzDREB45*, *DzDREB62*, and *DzDREB66* showed comparatively stronger support across datasets. These results provide candidate resources for further functional investigation of differential chilling responses in durian.

## 1. Introduction

Durian is a tropical evergreen tree belonging to the genus *Durio* in the family Malvaceae. It is an economically important fruit crop in Thailand, Malaysia, and Indonesia [1]. Its optimal growth temperature ranges from 25 to 32 °C, and it generally requires a relative humidity of 50–80%, making it well-suited to warm, frost-free tropical environments [2]. In China, commercial durian cultivation is concentrated in the southern part of Hainan Island, and episodic chilling events represent an important environmental constraint on seedling establishment, vegetative growth, and the northward expansion of cultivation.

Importantly, cultivar-dependent differences in chilling tolerance have recently been reported in durian. In a comparative study of four durian cultivars exposed to 10 °C, integrated evaluation of leaf anatomical traits and photosynthetic characteristics ranked chilling tolerance as ‘Kanyao’ > ‘Musang King’ > ‘Black Thorn’ > ‘Monthong’ [3]. This independent evidence indicates that ‘Musang King’ is relatively more chilling-tolerant than ‘Monthong’ and provides a biological basis for comparing their physiological and transcriptional responses to chilling.

Chilling stress disrupts membrane integrity, increases electrolyte leakage and lipid peroxidation, and promotes the excessive production of reactive oxygen species [4,5]. Plants respond by activating antioxidant enzymes, accumulating compatible osmolytes, and adjusting carbon metabolism and photosynthetic pigment composition [4,5]. These responses are highly dynamic and may differ among cultivars and developmental stages [5]. However, the temporal physiological responses of durian seedlings to prolonged chilling remain poorly characterized, and the relationship between visible injury, membrane damage, osmotic adjustment, and antioxidant capacity has not been systematically evaluated.

The AP2/ERF superfamily is one of the largest groups of plant-specific transcription factors and plays central roles in development and abiotic stress responses [6]. DREB proteins contain a conserved AP2 DNA-binding domain and recognize dehydration-responsive element/C-repeat motifs in the promoters of stress-responsive genes. Based on phylogenetic relationships, plant DREB proteins are commonly classified into six subgroups, A-1 to A-6. Members of the A-1 subgroup, also known as CBF/DREB1 proteins, are key components of the canonical ICE–CBF–COR pathway [7,8,9], whereas members of the other subgroups participate in diverse responses to dehydration, salinity, heat, and low temperature [10]. Genome-wide DREB analyses have been reported in several tropical fruit crops, including banana, jackfruit, pineapple, mango, and litchi, but a systematic analysis of the DREB family in durian is still lacking.

Based on the previously reported difference in chilling tolerance between ‘Musang King’ and ‘Monthong’, we hypothesized that the relatively chilling-tolerant ‘Musang King’ would exhibit more sustained or preferential transcriptional activation of a subset of stress-responsive genes during chilling. We therefore characterized the temporal phenotypic and physiological responses of the two cultivars during exposure to 10 °C and subsequently used comparative transcriptome analysis to construct a Musang King-focused discovery set of stress-responsive genes. Because several AP2/ERF transcription factors emerged from this analysis and DREB proteins are widely involved in abiotic-stress responses, we further performed a genome-wide characterization of the entire durian DREB family. Selected DzDREB candidates were then examined using exploratory seasonal field-expression profiling. The study aimed to prioritize DzDREB genes potentially associated with differential chilling responses between the two cultivars for subsequent functional investigation.

## 2. Results

### 2.1. Leaf Phenotypic Responses to Chilling

**Figure 1:**
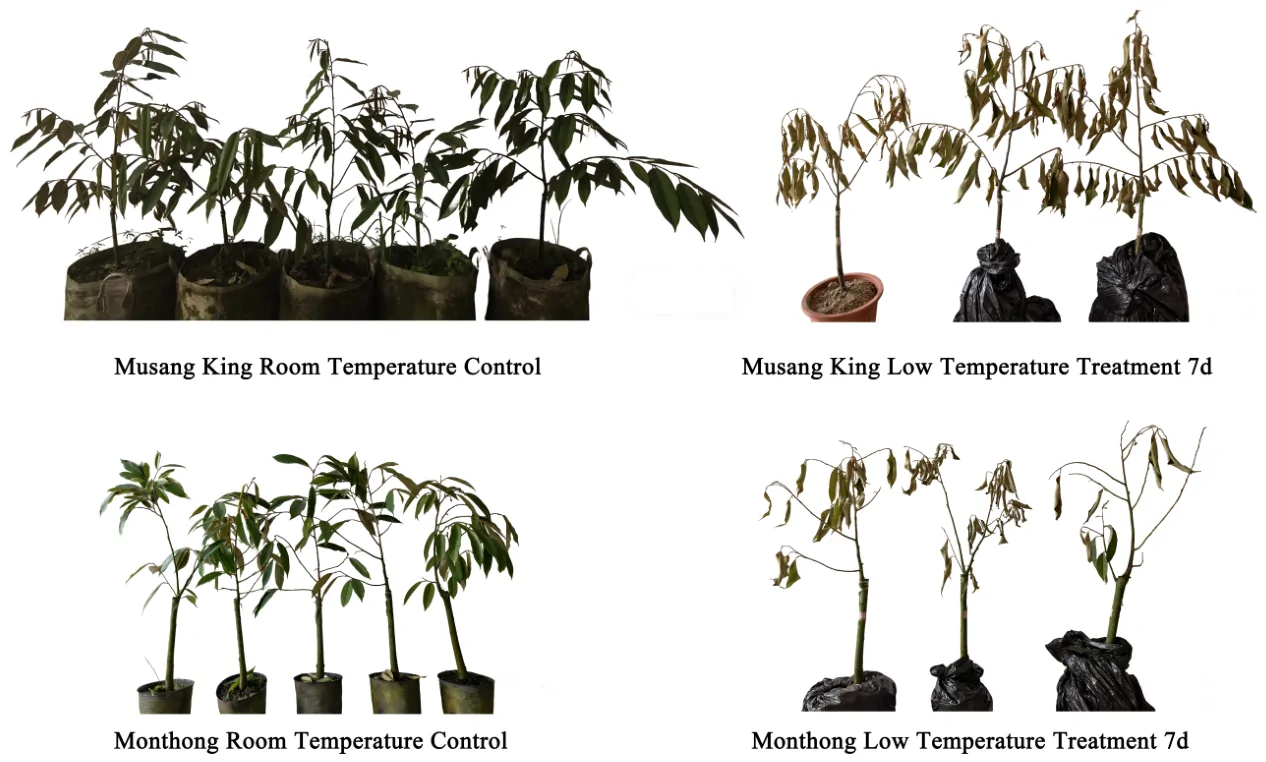
Representative phenotypes of ‘Musang King’ and ‘Monthong’ durian seedlings before chilling treatment and after 7 days of exposure to 10 °C. Three seedlings per cultivar were included as biological replicates in the controlled chilling experiment. Representative plants are shown; two additional untreated reserve seedlings are visible in the control image but were not included as biological replicates in the reported analyses.

Seedlings of both cultivars developed visible chilling injury during exposure to 10 °C (Figure 1). Based on daily visual observations, leaf abscission was first observed in ‘Monthong’ on day 1, whereas obvious leaf abscission in ‘Musang King’ was first observed on day 3. As chilling continued, leaf loss became increasingly apparent in both cultivars and was visually more extensive in ‘Monthong’. By day 7, both cultivars exhibited pronounced leaf injury and defoliation. Thus, ‘Monthong’ showed an earlier onset and visually greater extent of chilling injury under the present experimental conditions.

### 2.2. Changes in Membrane Injury, Osmotic Adjustment, Pigment Contents, and Enzyme Activities

**Figure 2:**
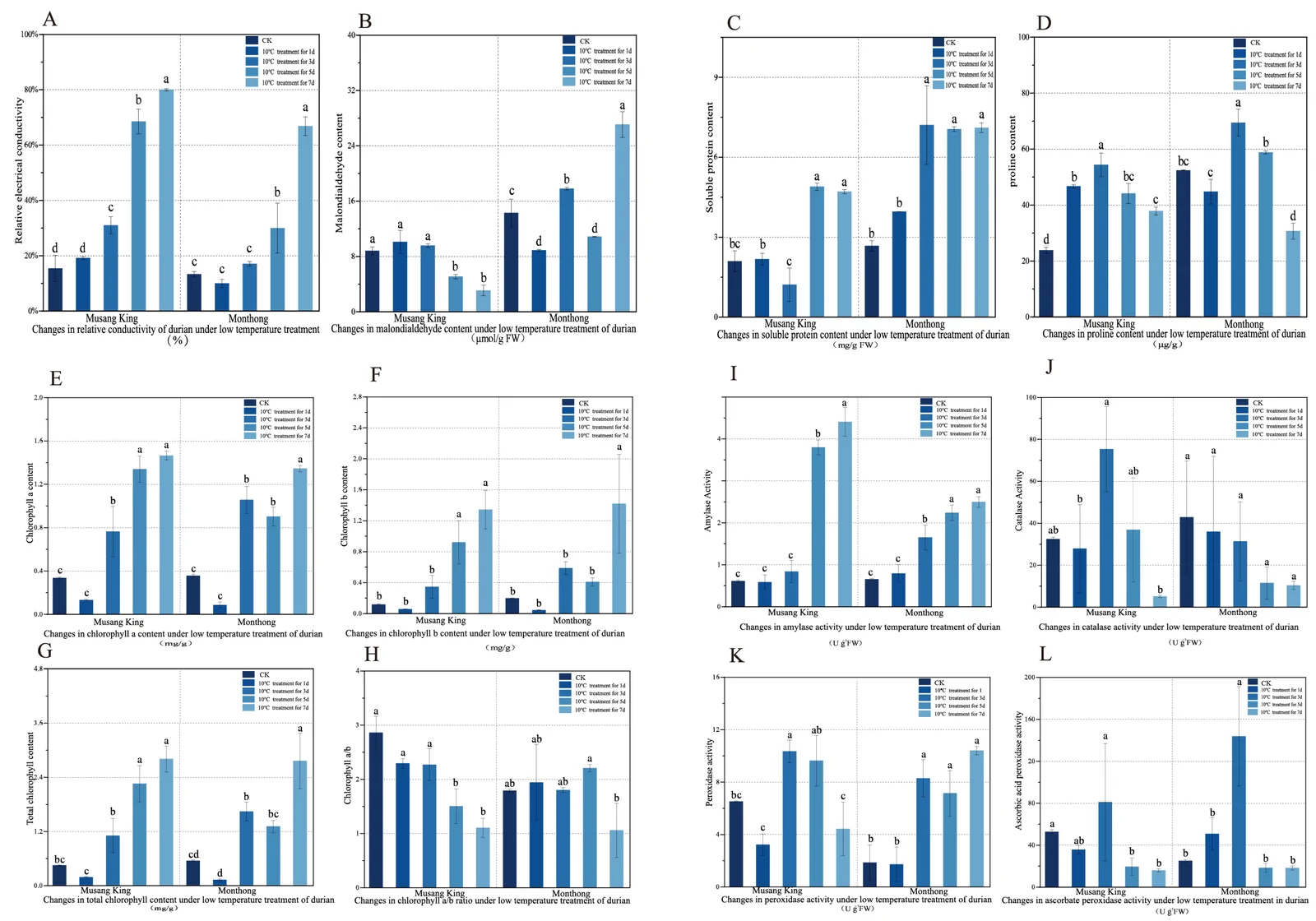
Physiological responses of durian leaves to chilling treatment. (**A**) Relative electrical conductivity; (**B**) malondialdehyde content; (**C**) soluble protein content; (**D**) proline content; (**E**) chlorophyll a; (**F**) chlorophyll b; (**G**) total chlorophyll; (**H**) chlorophyll a/b ratio; (**I**) amylase activity; (**J**) catalase activity; (**K**) peroxidase activity; and (**L**) ascorbate peroxidase activity. Data are presented as mean ± SD (*n* = 3 biological replicates). CK represents the pretreatment control sampled before initiation of the chilling treatment. Different lowercase letters above the bars indicate significant differences among treatments within the same cultivar (*p* < 0.05).

Relative electrical conductivity showed an overall increasing trend with chilling duration in both cultivars, indicating progressively increased membrane permeability relative to the pretreatment condition (Figure 2A). MDA content in ‘Musang King’ increased during the early stage of chilling and subsequently declined after day 3, whereas that in ‘Monthong’ showed an overall increasing pattern and reached its highest measured value on day 7 (Figure 2B). These two cultivars therefore exhibited contrasting temporal patterns of MDA accumulation during prolonged chilling.

Proline content increased during the early stage of chilling in both cultivars, reached its highest measured value on day 3, and subsequently declined (Figure 2D). Soluble protein content also increased relative to the pretreatment level, reaching its highest measured value on day 5 in ‘Musang King’ and on day 3 in ‘Monthong’, followed by stabilization or a slight decline (Figure 2C).

Relative to the pretreatment control, chlorophyll a, chlorophyll b, and total chlorophyll contents decreased after 1 day of chilling and subsequently showed an increasing trend during prolonged treatment, reaching their highest measured values on day 7 (Figure 2E–G). The chlorophyll a/b ratio generally declined with treatment duration and reached its lowest measured value on day 7 in both cultivars (Figure 2H).

Antioxidant enzyme activities showed different temporal patterns between the two cultivars. In ‘Musang King’, POD, CAT, and APX activities increased during the early stage of chilling, generally reached their highest values around day 3, and subsequently declined. In ‘Monthong’, APX activity initially increased and then decreased, and CAT activity progressively declined, whereas POD activity continued to increase through day 7. Amylase activity increased with treatment duration in both cultivars (Figure 2I–L). Among the measured physiological traits, MDA accumulation and antioxidant enzyme activities displayed particularly contrasting temporal patterns between ‘Musang King’ and ‘Monthong’.

### 2.3. Comparative Transcriptome Analysis and Construction of a Musang King-Focused Discovery Set

Transcriptome sequencing was performed using 18 leaf samples representing two cultivars, three treatment stages, and three biological replicates per treatment. A total of 109.17 Gb of raw sequencing data was generated (Supplementary Table S1). After quality filtering, individual libraries contained approximately 35.3–49.5 million clean reads, with Q20 values above 97% and Q30 values ranging from 92.02% to 94.55%. The GC content was approximately 42.4–44.1%. Clean-read mapping rates against the durian reference genome ranged from 91.61% to 95.73%, indicating successful alignment of the RNA-seq reads to the reference genome.

Principal component analysis showed that PC1 and PC2 explained 48.81% and 24.71% of the total expression variation, respectively. Most biological replicates clustered closely within their respective groups; however, the second biological replicate of ‘Monthong’ at day 5 (MT5d_2) showed greater separation from the other two MT5d replicates than was observed for most other replicate sets. Sample-correlation analysis showed generally high correlations among biological replicates, although the MT5d group exhibited somewhat greater within-group variability. All three MT5d biological replicates were retained for downstream analyses.

Differential expression was assessed using the predefined criterion of *p* < 0.05 and |log2FC| ≥ 1. To construct a Musang King-focused discovery set of genes showing progressive transcriptional activation in ‘Musang King’ during chilling, genes significantly up-regulated after 1 day relative to the corresponding pretreatment control were intersected with genes showing further significant up-regulation from day 1 to day 5. The same procedure was independently applied to ‘Monthong’. This analysis identified 2807 genes in ‘Musang King’ and 493 genes in ‘Monthong’ that met the progressive-up-regulation criterion. For construction of the Musang King-focused discovery set, genes that also fulfilled the same progressive-up-regulation criterion in ‘Monthong’ were not retained, leaving 2662 genes.

In parallel, genes showing higher transcript abundance in ‘Musang King’ than in ‘Monthong’ at day 1 were intersected with those showing higher transcript abundance in ‘Musang King’ at day 5, yielding 984 genes. Intersecting this set with the 2662-gene Musang King-focused progressive-response set yielded a 331-gene discovery set for downstream transcription-factor family prioritization (Supplementary Figure S1).

NR annotation of the 331-gene discovery set revealed genes encoding late embryogenesis abundant proteins, heat shock proteins, and several transcription-factor families, including AP2/ERF, bHLH, NAC, MYB, and C2H2-type zinc-finger proteins. Because DREB proteins constitute a major stress-responsive subgroup of the AP2/ERF family and have established roles in plant responses to low temperature and other abiotic stresses, the DREB family was selected for systematic genome-wide characterization.

### 2.4. Identification and Expression Analysis of the DREB Gene Family in Durian

The initial 331-gene Musang King-focused set was used as a discovery step to identify transcription-factor families warranting further investigation. After the DREB family was selected for systematic analysis, all 81 DzDREB members were evaluated at the family-wide level, irrespective of whether individual members were included in the initial 331-gene set. This broader analysis was adopted because DzDREB genes responsive in either or both cultivars could potentially participate in the general chilling-response network. We therefore performed genome-wide identification, phylogenetic classification, structural and syntenic analyses, promoter analysis, functional annotation, and expression profiling of the entire DzDREB family, followed by candidate prioritization.

#### 2.4.1. Identification and Multiple Sequence Alignment of the DREB Gene Family in Durian

A total of 247 AP2/ERF (Supplementary Table S2) superfamily members were identified in the durian genome using complementary BLASTP and HMM searches in TBtools v2.326. Based on domain composition and conserved amino acid residues, these proteins were classified into four major groups. Thirty-five proteins containing two AP2 domains were assigned to the AP2 subfamily, and eight proteins containing one AP2 domain and one B3 domain were assigned to the RAV subfamily. Among the proteins containing a single AP2 domain, 123 were classified as ERF proteins and 81 as DREB proteins according to the characteristic amino acid residues at positions 14 and 19 of the AP2 domain (Supplementary Figure S2). The 81 DREB genes were designated *DzDREB1*–*DzDREB81* according to their chromosomal positions (Supplementary Figure S3).

#### 2.4.2. Physicochemical Properties of the Durian DREB Proteins

Supplementary Table S3 summarizes the physicochemical properties of the 81 durian DREB proteins. The DzDREB proteins ranged from 149 to 1055 amino acids in length, with predicted molecular masses varying between 16.21 and 117.14 kDa. The theoretical isoelectric points (pI) spanned 4.4–9.76; of these, 59 members (73%) had pI values below 7 (acidic), 21 (26%) above 7 (basic), and one protein had a predicted pI of 7.00. Seventy-five of the 81 proteins were predicted to be unstable. The aliphatic index values were distributed from 47.44 to 90.38, and hydropathy analysis revealed that all proteins except DzDREB49 were hydrophilic. Subcellular localization analysis predicted that 72 DzDREB proteins were localized to the nucleus, whereas the remaining proteins were assigned to the cytoplasm (3), mitochondria (1), chloroplasts (4), or plasma membrane (1).

#### 2.4.3. Phylogenetic Analysis of the DREB Gene Family in Durian

**Figure 3:**
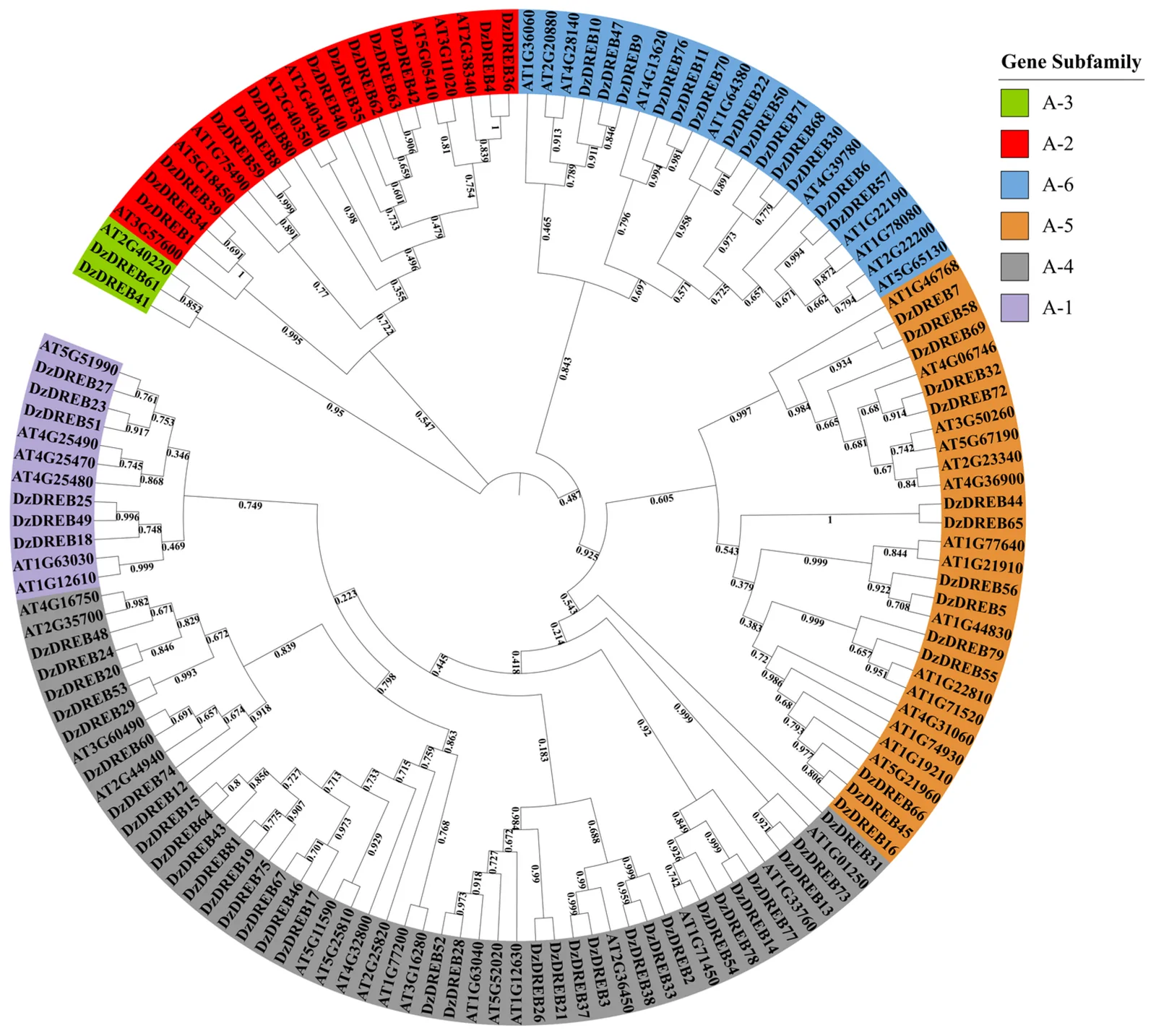
Phylogenetic Tree and Subgroup Classification of DREB Proteins in Durian and *Arabidopsis*.

A phylogenetic tree was constructed using DREB family members from durian and *Arabidopsis* (Figure 3). According to the classification of DREB protein subfamilies in *Arabidopsis*, the DzDREB proteins were divided into six subgroups: A-1, A-2, A-3, A-4, A-5, and A-6. Among them, subgroup A-4 contained the largest number of members (33), while subgroup A-1, A-2, A-5, and A-6 contained 6, 13, 14, and 13 members, respectively, and subgroup A-3 contained the fewest members (2). The distribution of the durian DREB proteins among the six subgroups was broadly consistent with the established *Arabidopsis* DREB classification. The clustering of durian and *Arabidopsis* proteins within the same subgroups is consistent with conservation of the major DREB lineages.

#### 2.4.4. Gene Structure, Conserved Domain, and Conserved Motif Analysis of the DREB Gene Family in Durian

**Figure 4:**
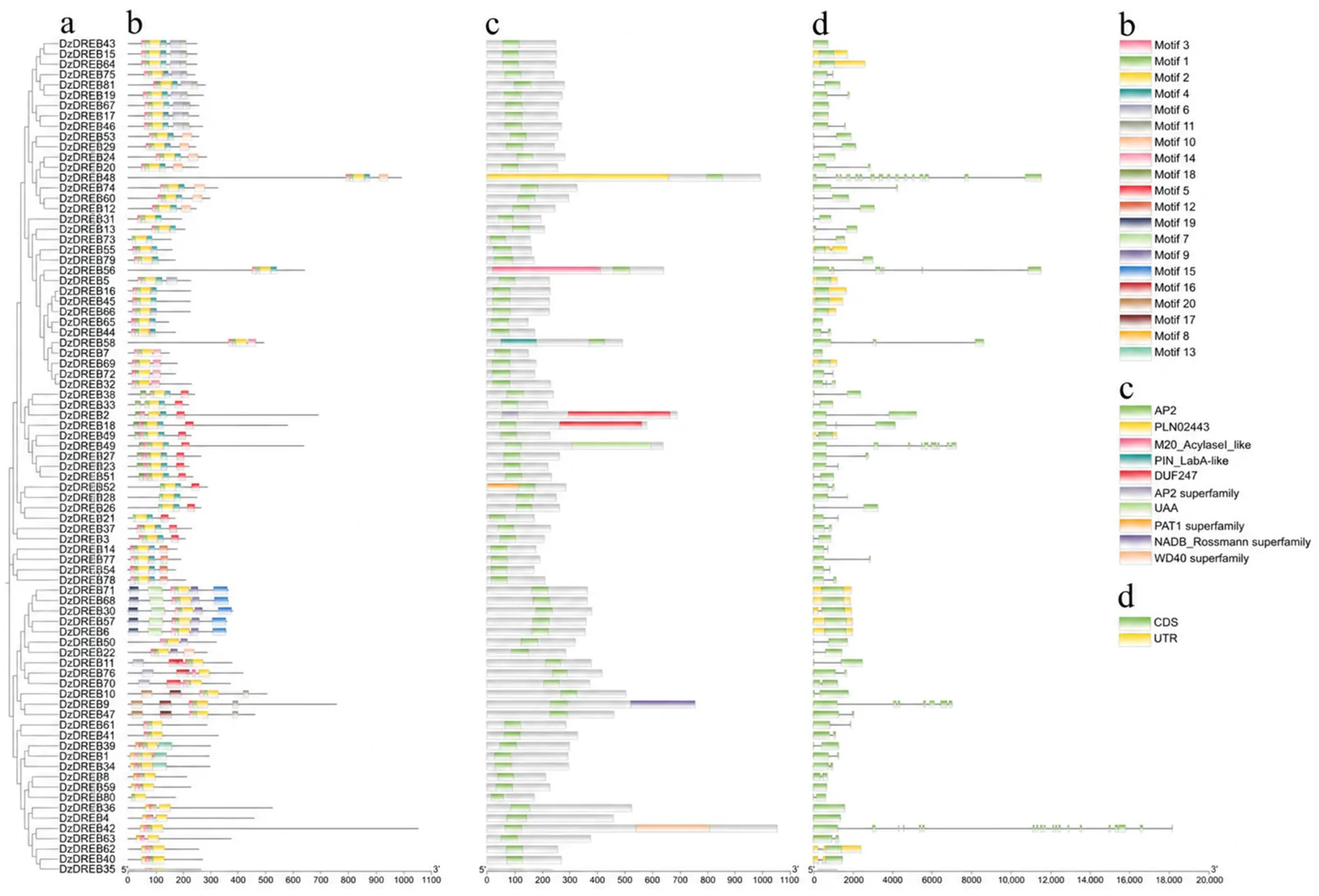
The phylogenetic tree (**a**), conserved motifs (**b**), protein domains (**c**), and gene structures (**d**) of DzDREB genes.

A total of 20 motifs (motif1–motif20) were identified in this study (Figure 4b). Conserved domain analysis of the DzDREB proteins (Figure 4c) revealed that all members contained the AP2 domain. Members clustering within the same phylogenetic subgroup generally displayed similar motif compositions and gene structures, whereas clear differences were observed among different subgroups.

#### 2.4.5. Promoter cis-Regulatory Element Analysis of the DREB Gene Family in Durian

**Figure 5:**
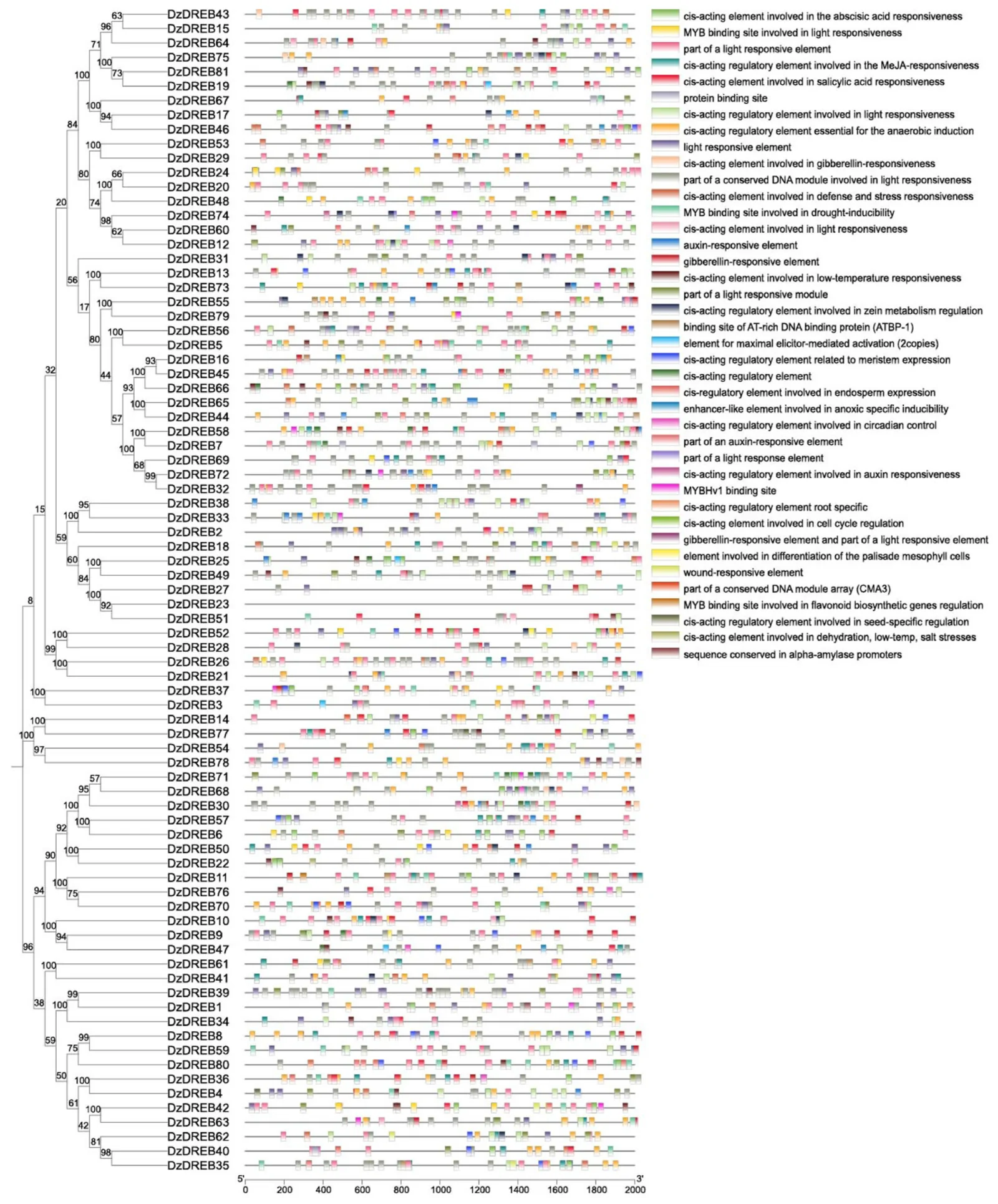
Distribution of cis-regulatory elements in the promoters of DzDREB genes.

A total of 40 types of cis-regulatory elements were detected in the 2 kb promoter regions of the 81 DzDREB genes (Figure 5). Among the stress-related elements, LTR motifs were detected in 29 promoters, DRE motifs in one promoter, TC-rich repeats in 36 promoters, and ABRE elements in 67 promoters. Among these stress-related elements, ABRE showed the widest distribution, occurring in 67 of the 81 DzDREB promoters, whereas DRE motifs were detected in only one promoter, revealing a marked difference in the prevalence of these two stress-related cis-regulatory elements.

#### 2.4.6. Chromosomal Distribution and Synteny Analysis of the DREB Gene Family in Durian

**Figure 6:**
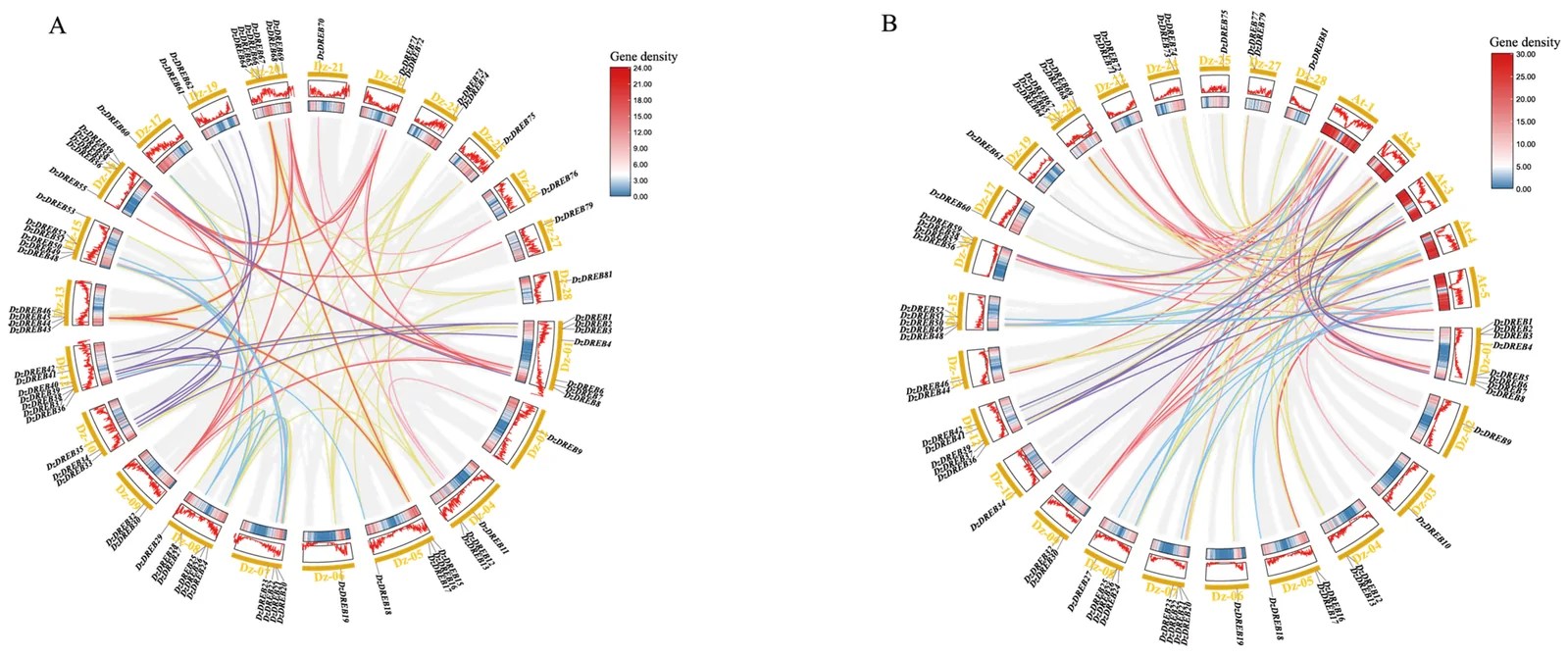
Synteny analysis of the DREB gene family in durian. (**A**) Intraspecies synteny analysis of DzDREB genes within the durian genome. (**B**) Interspecies synteny analysis between durian and *Arabidopsis* DREB gene families.

The chromosomal locations of DzDREB family members were analyzed (Supplementary Figure S3). The 81 DzDREB genes were unevenly distributed across 25 chromosomes. Intragenomic collinearity analysis identified 92 syntenic DzDREB gene pairs (Figure 6A), indicating extensive collinear relationships among members of the family. Comparative synteny analysis identified 119 syntenic relationships between durian and *Arabidopsis thaliana* DREB loci (Figure 6B), supporting conservation of multiple DREB-associated genomic regions between the two species.

#### 2.4.7. GO and KEGG Enrichment Analyses of the DzDREB Gene Family

**Figure 7:**
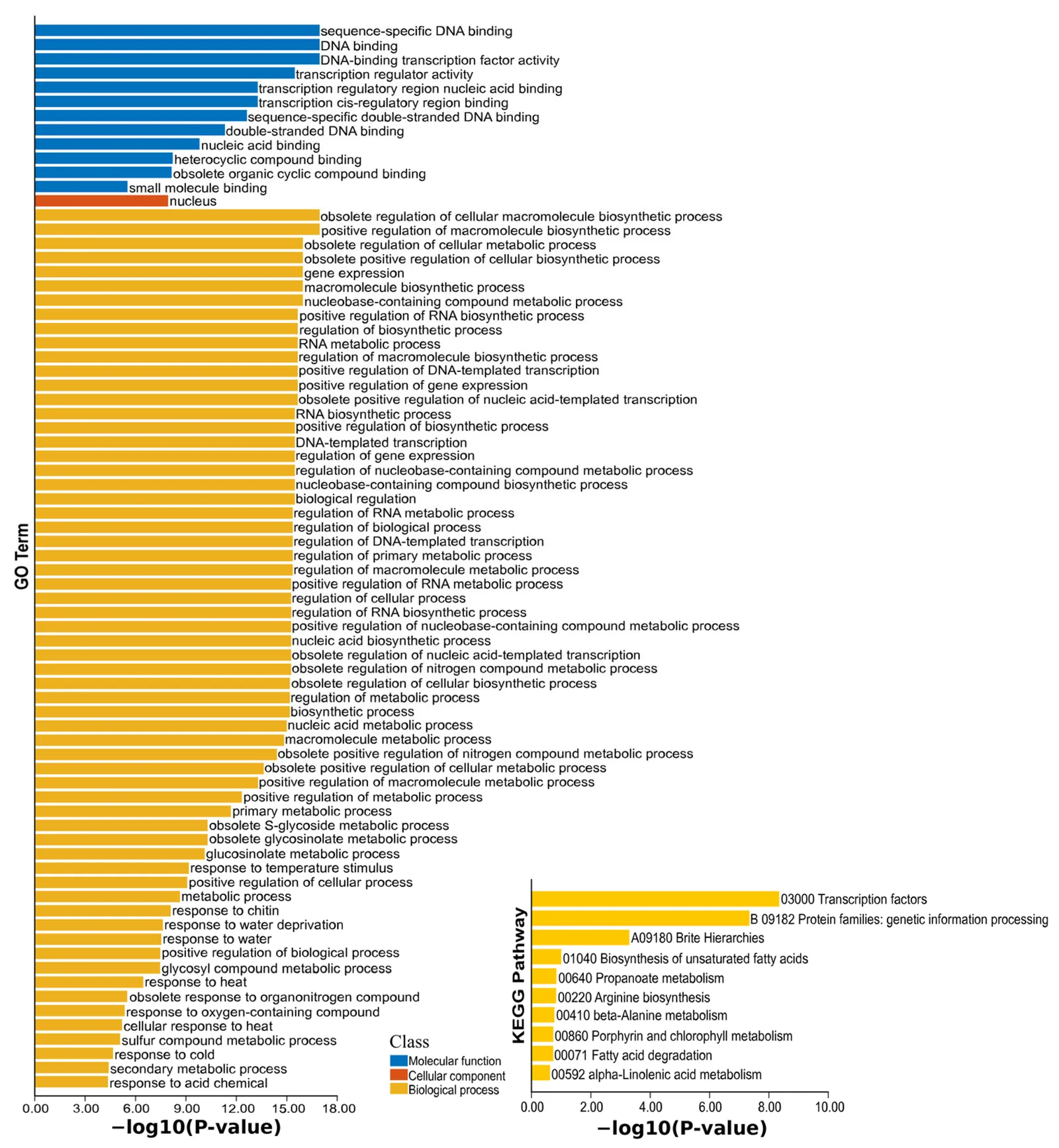
GO and KEGG enrichment analyses of the durian DREB gene family.

GO and KEGG enrichment analyses were performed for the 81 DzDREB genes using a hypergeometric test (Figure 7). Within the biological process category, 11 genes were associated with “response to cold”, 15 with “response to water deprivation”, and 19 with “response to temperature stimulus”. Seven genes—*DzDREB6*, *DzDREB7*, *DzDREB23*, *DzDREB25*, *DzDREB27*, *DzDREB51*, and *DzDREB57*—were common to all three GO terms.

KEGG enrichment analysis assigned DzDREB members mainly to the categories “Transcription factors”, “Protein families: genetic information processing”, and “BRITE hierarchies”.

#### 2.4.8. Candidate Prioritization and Exploratory Seasonal Field-Expression Profiling of Selected DzDREB Genes

**Figure 8:**
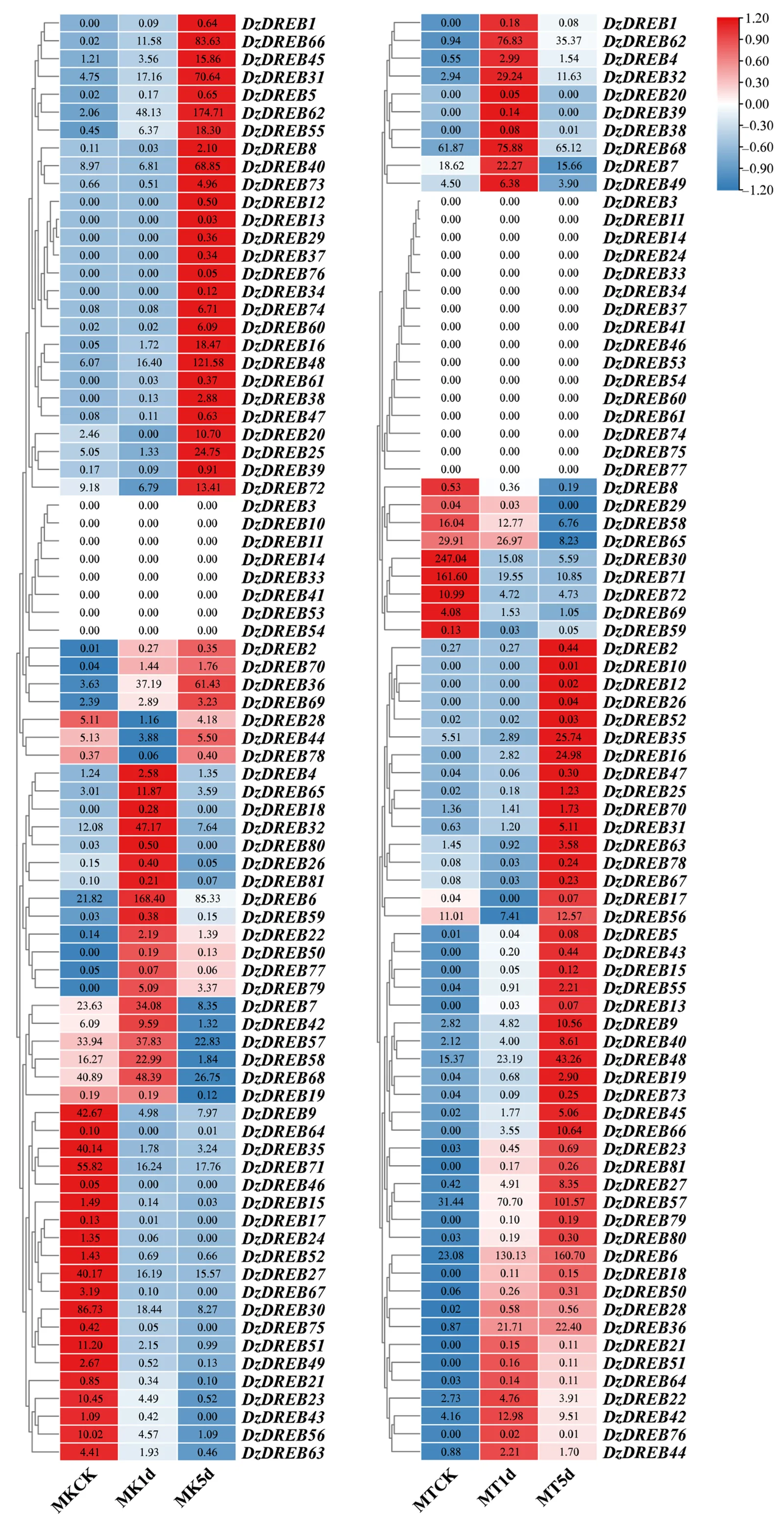
Expression profiles of DzDREB genes in ‘Musang King’ and ‘Monthong’ under chilling stress. Colors represent row-scaled Z-scores, whereas the values displayed in the cells represent mean FPKM values from three biological replicates. MKCK and MTCK represent the corresponding pretreatment control samples; MK1d, MK5d, MT1d, and MT5d represent samples collected after 1 and 5 days of chilling treatment, respectively.

Expression profiles of all 81 DzDREB genes were first examined using the controlled-environment transcriptome dataset (Figure 8). In ‘Musang King’, 30 genes were up-regulated and 21 were down-regulated after 1 day of chilling relative to the pretreatment control. Eleven genes—*DzDREB2*, *DzDREB16*, *DzDREB31*, *DzDREB36*, *DzDREB45*, *DzDREB48*, *DzDREB55*, *DzDREB62*, *DzDREB66*, *DzDREB69*, and *DzDREB70*—showed progressively increasing mean transcript abundance from the pretreatment stage through day 1 to day 5. In ‘Monthong’, 29 genes were up-regulated and 11 were down-regulated after 1 day, and 20 genes showed progressively increasing mean transcript abundance across the sampled chilling stages.

To prioritize DzDREB genes for further analysis, all 81 family members were evaluated using three complementary categories of evidence rather than a single mandatory inclusion rule: (i) transcriptomic response patterns during the controlled chilling experiment, including progressive expression changes and differential-expression status; (ii) annotation to stress-related GO terms, particularly “response to cold”, “response to temperature stimulus”, and “response to water deprivation”; and (iii) the occurrence of stress-related promoter cis-regulatory elements, including LTR, DRE, TC-rich repeats, and ABRE. These evidence categories were considered jointly for candidate prioritization and were not required to be simultaneously satisfied by every selected gene. Based on an integrative evaluation of these complementary evidence categories, ten genes—*DzDREB6*, *DzDREB7*, *DzDREB25*, *DzDREB36*, *DzDREB42*, *DzDREB45*, *DzDREB48*, *DzDREB57*, *DzDREB62*, and *DzDREB66*—were prioritized as a non-exhaustive panel for subsequent qRT-PCR analysis. The evidence profiles and candidate status of all 81 DzDREB genes are summarized in Supplementary Table S5.

**Figure 9:**
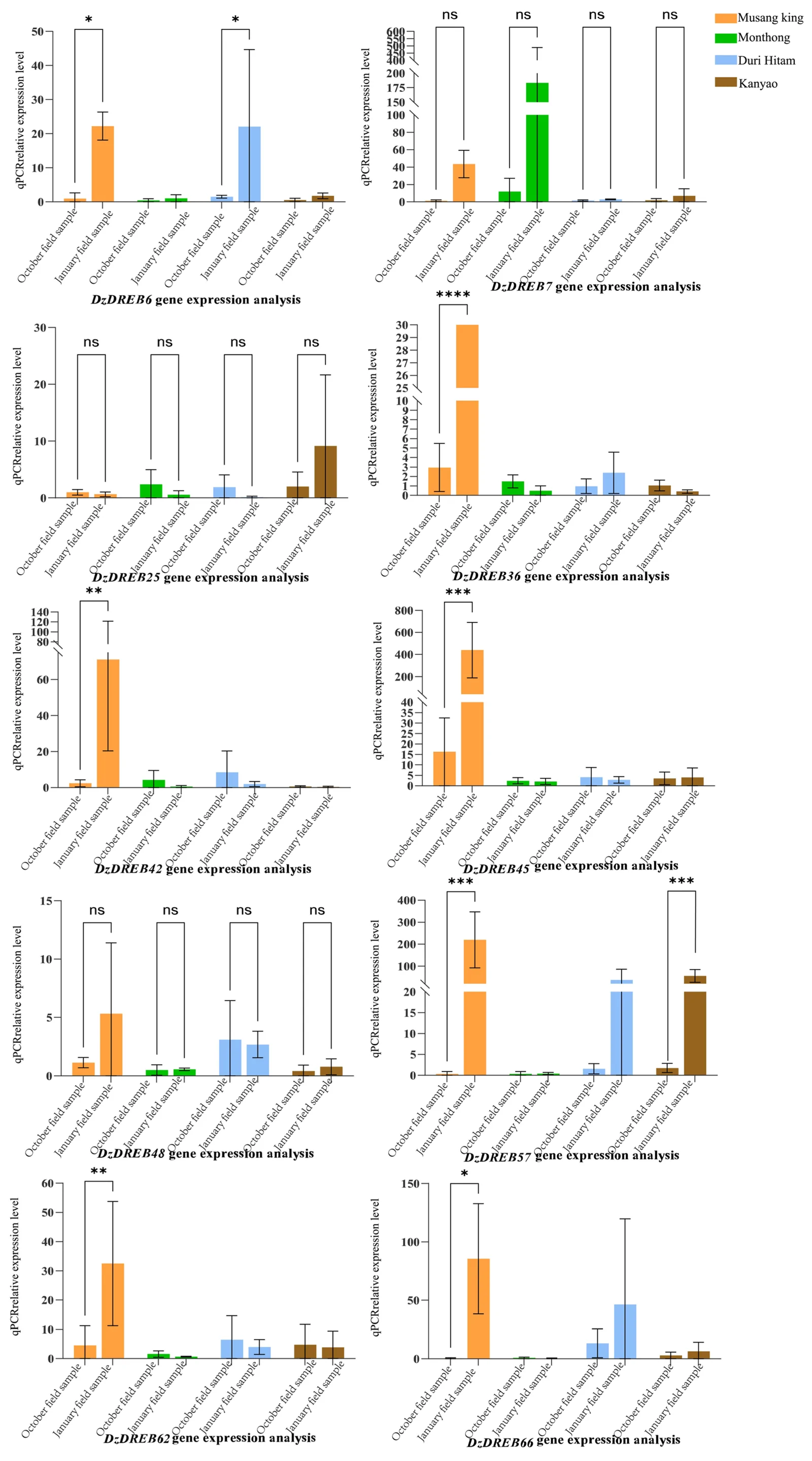
Relative transcript abundance of selected DzDREB genes in four durian cultivars under contrasting autumn and winter field conditions. October and January samples were collected from the same orchard but differed in temperature, sampling time, season, photoperiod, and humidity. Data are presented as the mean ± SD of three biological replicates. * *p* < 0.05, ** *p* < 0.01, *** *p* < 0.001, and **** *p* < 0.0001; ns, not significant.

Exploratory seasonal field-expression profiling was then performed for the ten prioritized genes (Figure 9). In ‘Musang King’, seven genes—*DzDREB6*, *DzDREB36*, *DzDREB42*, *DzDREB45*, *DzDREB57*, *DzDREB62*, and *DzDREB66*—showed significantly higher transcript abundance in January than in October, whereas *DzDREB7* and DzDREB48 showed increasing tendencies and *DzDREB25* showed a decreasing tendency. In ‘Monthong’, four genes showed increasing tendencies; in ‘Duri Hitam’, one gene showed a significant seasonal increase and four showed increasing tendencies; and in ‘Kanyao’, one gene showed a significant seasonal increase and six showed increasing tendencies.

The seasonal field-expression data were used as supplementary evidence for the ten prioritized genes and did not alter the ten-gene panel. Among them, *DzDREB36*, *DzDREB45*, *DzDREB62*, and *DzDREB66* also showed progressive induction during the controlled chilling experiment and therefore received comparatively stronger support across datasets. The remaining candidates were retained based on the controlled-environment transcriptomic, functional-annotation, and promoter evidence.

## 3. Discussion

### 3.1. Temporal Patterns of Membrane Injury, Osmotic Adjustment, and Pigment Changes During Chilling

Previous comparative evaluation of durian cultivars under 10 °C chilling ranked ‘Musang King’ above ‘Monthong’ in overall chilling tolerance [3]. Consistent with this previously reported cultivar difference, ‘Monthong’ exhibited an earlier onset and visually greater extent of leaf abscission in the present experiment. The two cultivars also showed contrasting MDA trajectories: MDA content in ‘Musang King’ increased during the early stage and subsequently declined, whereas that in ‘Monthong’ continued to increase and reached its highest measured level on day 7. Because MDA is widely used as an indicator of membrane lipid peroxidation, these patterns are consistent with differential progression of oxidative membrane injury during prolonged chilling [11,12,13]. Together, the phenotypic observations and MDA dynamics provide physiological evidence consistent with the previously reported difference in chilling tolerance between the two cultivars.

Chlorophyll a, chlorophyll b, and total chlorophyll contents showed a non-linear temporal pattern, with an initial decrease after 1 day followed by an apparent increase during prolonged chilling. Because chlorophyll contents were expressed on a fresh-weight basis, progressive leaf dehydration and the associated reduction in tissue water content could have increased the measured chlorophyll concentration per unit fresh weight without necessarily reflecting an increase in absolute chlorophyll content. In addition, progressive leaf abscission may have preferentially removed severely injured leaves, leaving relatively fewer injured leaves available for sampling at later stages. Therefore, the late-stage increase should be interpreted cautiously as an apparent concentration increase rather than evidence of physiological recovery.

Proline content increased during the early stage of chilling and reached a maximum at day 3 before declining in both cultivars. Such transient proline accumulation is consistent with its widely reported role in osmotic adjustment and cellular protection during abiotic stress [14,15]. Soluble protein content showed a somewhat different temporal pattern, with later maxima in ‘Musang King’ than in ‘Monthong’. Amylase activity increased progressively during chilling in both cultivars, indicating an enhanced carbohydrate-mobilization response under prolonged low-temperature conditions. Increased amylase activity and starch degradation have similarly been associated with soluble-sugar mobilization during plant responses to low temperature [16,17]. Together, these changes reflect dynamic adjustments in osmotic regulation and carbohydrate-related metabolism during chilling.

### 3.2. Temporal Patterns of Antioxidant Enzyme Responses in the Two Cultivars

Antioxidant enzyme activities showed distinct temporal profiles between the two cultivars. In ‘Musang King’, POD, CAT, and APX activities increased during the early stage of chilling and generally reached their highest levels around day 3, whereas ‘Monthong’ exhibited a progressive decline in CAT activity but a continued increase in POD activity. When considered together with the contrasting MDA trajectories, the coordinated early changes in antioxidant enzyme activities in ‘Musang King’ indicate a more pronounced early antioxidant response, which is consistent with previous observations that genotypes differing in chilling tolerance can exhibit distinct antioxidant responses under low-temperature stress [11,12].

### 3.3. Structural and Evolutionary Characteristics of the Durian DREB Gene Family

A total of 81 DzDREB genes were identified in the durian genome and classified into six subgroups. The number of identified DREB genes differs among plant species, including banana (103) [18], jackfruit (93) [19], *Arabidopsis* (56) [20], pineapple (20) [21], mango (42) [22], and litchi (39) [23]. Such variation may reflect differences in lineage-specific gene duplication and loss, genome evolutionary history, genome assembly, annotation, and classification criteria [24]. The 92 intragenomic syntenic DzDREB gene pairs further demonstrate extensive collinear relationships within the durian DREB family, while the 119 syntenic relationships identified between durian and *Arabidopsis* support evolutionary conservation of multiple DREB-associated genomic regions.

Promoter analysis revealed a striking contrast in the distribution of ABRE and DRE elements among DzDREB genes. ABRE elements were detected in 67 of the 81 promoters, suggesting that ABA-associated signaling may represent one potential component of the upstream regulation of the DzDREB family [25]. In contrast, a DRE motif was detected in only one DzDREB promoter. This scarcity does not necessarily imply a limited role of DzDREB genes in chilling responses, because DREB proteins primarily function by recognizing DRE/CRT elements in the promoters of downstream stress-responsive genes rather than requiring DRE motifs within their own promoters [20,26]. The low frequency of promoter DRE motifs may therefore indicate that widespread DRE-mediated autoregulatory or cross-regulatory inputs are not a dominant feature of DzDREB transcriptional regulation [27]. Instead, their transcription may depend on other upstream regulatory signals, potentially including ABA-associated and other stress-responsive pathways. Thus, the contrasting distribution of ABRE and DRE elements highlights the distinction between the upstream regulation of DzDREB genes and the downstream DNA-binding functions of the encoded DREB transcription factors.

### 3.4. Prioritization of DzDREB Candidates Associated with Differential Chilling Responses

The comparative transcriptome analysis was designed to construct a Musang King-focused discovery set based on the previously reported higher chilling tolerance of ‘Musang King’ relative to ‘Monthong’. The initial 331-gene set served as a discovery dataset for identifying transcription-factor families of interest. After the DREB family was selected, all 81 DzDREB members were reconsidered at the family-wide level, allowing both cultivar-biased and shared chilling-responsive DzDREB genes to be incorporated into the subsequent analysis.

The two cultivars exhibited distinct DzDREB expression profiles during chilling. For family-wide candidate prioritization, transcriptomic response patterns during the controlled chilling experiment were considered together with stress-related GO annotations and promoter cis-regulatory elements. These three evidence categories were treated as complementary rather than as mandatory criteria that every selected gene had to satisfy simultaneously. Accordingly, different combinations of transcriptomic, functional-annotation, and promoter evidence were considered across the DzDREB family. On this basis, a non-exhaustive ten-gene panel comprising *DzDREB6*, *DzDREB7*, *DzDREB25*, *DzDREB36*, *DzDREB42*, *DzDREB45*, *DzDREB48*, *DzDREB57*, *DzDREB62*, and *DzDREB66* was prioritized for subsequent qRT-PCR analysis. Similar combinations of genome-wide DREB-family characterization and stress-responsive expression profiling have been used to prioritize candidate DREB genes associated with cold responses in other tropical fruit crops, including banana and jackfruit [18,19].

Seasonal field-expression profiling provided additional support for seven of the ten candidates in ‘Musang King’. Among them, *DzDREB36*, *DzDREB45*, *DzDREB62*, and *DzDREB66* showed expression patterns consistent with their progressive induction in the controlled chilling experiment and therefore received comparatively stronger support across datasets. *DzDREB6*, *DzDREB42*, and *DzDREB57* also received additional seasonal expression support, while *DzDREB7*, *DzDREB25*, and *DzDREB48* remained within the ten-gene panel based on the integrated controlled-environment evidence. Thus, the combined analyses established a tiered set of DzDREB candidates for subsequent functional investigation.

The prioritized genes include members outside the canonical A-1/CBF subgroup, suggesting that durian chilling responses may involve broader DREB-mediated regulatory networks in addition to the classical ICE–CBF–COR module [9]. Although DREB1/CBF members are central components of the canonical cold-response pathway, DREB proteins from other subgroups have also been implicated in diverse abiotic-stress responses, including cold, drought, salinity, and heat [28,29]. Further characterization of their DNA-binding properties, downstream targets, and regulatory relationships will help clarify their specific functions in the durian chilling response.

## 4. Materials and Methods

### 4.1. Plant Materials

Twelve-month-old seedlings of two durian cultivars, ‘Musang King’ and ‘Monthong’, approximately 1 m in height, were purchased and used for the controlled chilling experiment, physiological measurements, and transcriptome sequencing. For each cultivar, three seedlings were assigned to the chilling treatment and three seedlings to the control condition.

For the exploratory field-expression analysis, leaves were collected from approximately five-year-old trees, approximately 4 m in height, of four durian cultivars: ‘Musang King’, ‘Monthong’, ‘Duri Hitam’, and ‘Kanyao’. The field trees were grown in the same orchard in Baoting Li and Miao Autonomous County, Hainan Province, China.

### 4.2. Controlled Chilling Treatment and Sample Collection

Plants assigned to the chilling treatment were maintained at 10 °C under a 12 h light/12 h dark photoperiod for 7 days, whereas control plants were maintained at 26 °C under the same 12 h light/12 h dark photoperiod. Relative humidity, light intensity/quality, airflow, and other environmental parameters were not recorded or experimentally matched between the two environments. The untreated plants maintained at 26 °C were used only for qualitative observation of leaf-abscission progression and were not included in physiological or transcriptomic analyses. A pretreatment control (CK) was sampled immediately before initiation of the chilling treatment and served as the single baseline for subsequent comparisons. Leaves from chilled plants were then collected after 1, 3, 5, and 7 days of treatment. Samples used for relative electrical conductivity measurements were analyzed immediately after collection, whereas samples for biochemical and molecular analyses were frozen in liquid nitrogen and stored at −80 °C. The pretreatment control and the 1- and 5-day chilling samples were used for RNA sequencing.

To explore the seasonal expression patterns of the prioritized DzDREB genes under orchard conditions, independent field samples were collected from the same orchard on 13 October 2025 and 9 January 2026. The October samples were collected at approximately 10:00 under 26 °C and 95% relative humidity, whereas the January samples were collected at approximately 06:00 under 12 °C and 86% relative humidity. Three biological replicates were collected for each cultivar, and each biological sample was analyzed using three technical qPCR replicates. Because the two sampling dates also differed in season, time of day, photoperiod, humidity, and developmental status, this comparison was treated as an exploratory seasonal field-expression analysis rather than a controlled validation of temperature effects.

### 4.3. Measurement of Physiological Indices

Pretreatment control leaves and leaves collected from plants exposed to 10 °C for 1, 3, 5, and 7 days were used for physiological measurements. The measured indices included relative electrical conductivity, malondialdehyde content, chlorophyll a content, chlorophyll b content, total chlorophyll content, the chlorophyll a/b ratio, proline content, soluble protein content, amylase activity, catalase activity, peroxidase activity, and ascorbate peroxidase activity. Each measurement was performed using three biological replicates.

#### 4.3.1. Determination of Relative Electrical Conductivity

Relative electrical conductivity (REC) was determined according to previously described electrolyte-leakage methods [30]. Healthy leaves were cut into approximately 1 cm × 1 cm squares. A 0.1 g sample was rinsed with tap water to remove surface contaminants and then washed two to three times with deionized water. After the surface moisture was removed using absorbent paper, the leaf pieces were transferred to a clean 50 mL centrifuge tube containing 10 mL of deionized water. The samples were incubated at room temperature for 30 min and gently shaken several times during incubation.

The initial electrical conductivity of the sample solution was measured and recorded as C1. The conductivity of deionized water was measured as the blank value, C0. The sample tubes were then boiled for 10 min to completely disrupt the leaf tissues, cooled to room temperature, mixed thoroughly, and measured again to obtain the final conductivity, C2. Relative electrical conductivity was calculated as follows:

REC (%) = [(C1 − C0)/(C2 − C0)] × 100,

where C0 is the conductivity of the deionized-water blank, C1 is the initial conductivity of the leaf incubation solution, and C2 is the conductivity after boiling.

#### 4.3.2. Determination of Chlorophyll Content

Fresh leaves were washed, blotted dry, cut into small pieces, and weighed to obtain 0.2 g of tissue. The samples were homogenized in a small volume of 95% ethanol (Guilin Lifeng Medical Supplies Co., Ltd., Guilin, China). The homogenate was transferred to a centrifuge tube, and the mortar was rinsed with 95% ethanol. The rinsing solution was combined with the homogenate, and the total extraction volume was recorded as V mL.

The homogenate was centrifuged at 3000–4000 rpm for 10 min. The absorbance of the supernatant was measured at 665 and 649 nm using 95% ethanol as the blank. Chlorophyll contents were calculated as follows:

Chlorophyll a (mg g^−1^ FW) = (13.95A665 − 6.88A649) × V/W

Chlorophyll b (mg g^−1^ FW) = (24.96A649 − 7.32A665) × V/W

Total chlorophyll (mg g^−1^ FW) = chlorophyll a + chlorophyll b

where A665 and A649 are the absorbance values at 665 and 649 nm, respectively, V is the total extraction volume, and W is the fresh weight of the sample [31].

#### 4.3.3. Determination of Malondialdehyde (MDA) Content

Fresh leaf tissue (0.2 g) was frozen in liquid nitrogen and ground into a fine powder. The powder was immediately homogenized with 2 mL of 10% trichloroacetic acid (Xilong Chemical Co., Ltd., Shantou, China). The homogenate was centrifuged at 8000 rpm for 10 min at 4 °C, and the supernatant was collected as the extract.

One milliliter of the extract was mixed with 1 mL of 0.6% thiobarbituric acid solution. For the blank, 1 mL of 10% trichloroacetic acid was mixed with 1 mL of 0.6% thiobarbituric acid solution. The mixtures were heated in a boiling-water bath for 15 min, rapidly cooled, and centrifuged at 6800 rpm for 10 min at 4 °C. The absorbance of the supernatant was measured at 450, 532, and 600 nm [32]. Malondialdehyde content was calculated as follows:

MDA content (μmol g^−1^ FW) = [6.45(A532 − A600) − 0.56A450] × V/W,

where V is the total extraction volume in mL and W is the fresh weight of the sample in g.

#### 4.3.4. Determination of Proline Content

Proline content was determined using the acid-ninhydrin colorimetric method described, with minor modifications [33]. Fresh leaf tissue (0.2 g) was frozen in liquid nitrogen and ground into a fine powder. The powder was immediately homogenized with 2 mL of 3% sulfosalicylic acid, mixed thoroughly, and centrifuged at 8000 rpm for 10 min.

One milliliter of the supernatant was mixed with 1 mL of glacial acetic acid (Guangzhou Chemical Reagent Factory, Guangzhou, China) and 1 mL of ninhydrin reagent. The blank contained 1 mL of distilled water, 1 mL of glacial acetic acid, and 1 mL of ninhydrin reagent (Sangon Biotech (Shanghai) Co., Ltd., Shanghai, China). The mixtures were heated in a boiling-water bath for 40 min and then cooled to room temperature. Five milliliters of toluene (Xilong Chemical Co., Ltd., Shantou, China) was added, and the mixtures were vigorously shaken to extract the colored product. The upper toluene phase was collected, and its absorbance was measured at 520 nm.

A standard curve was constructed using proline solutions ranging from 0 to 20 μg mL^−1^. Proline content was calculated as follows:

Proline content (μg g^−1^ FW) = C × V/W,

where C is the proline concentration determined from the standard curve, V is the total extraction volume, and W is the fresh weight of the sample.

#### 4.3.5. Determination of Amylase Activity

Fresh leaf tissue (0.2 g) was frozen in liquid nitrogen and ground into a fine powder. The powder was immediately homogenized with 2 mL of 0.1 M phosphate buffer (pH 7.0). After centrifugation at 8000 rpm for 10 min at 4 °C, the supernatant was collected as the crude enzyme extract and maintained on ice.

A 0.5 mL aliquot of the crude enzyme extract was mixed with 0.5 mL of 1% soluble starch solution (Guangzhou Chemical Reagent Factory, Guangzhou, China) and incubated at 37 °C for 20 min. The reaction was terminated by adding 1 mL of DNS reagent, followed by boiling for 5 min. After cooling, the reaction mixture was adjusted to a final volume of 5 mL with distilled water. Absorbance was measured at 540 nm [34]. A mixture containing 4 mL of distilled water and 1 mL of DNS reagent was used as the blank.

A standard curve was constructed using glucose solutions ranging from 0 to 1 mg mL^−1^. Amylase activity was calculated as follows:

Amylase activity (U g^−1^ FW) = C × V × DF/(W × T),

where C is the glucose concentration determined from the standard curve, V is the total volume of the enzyme extract, DF is the dilution factor, W is the fresh weight of the sample, and T is the reaction time in min.

#### 4.3.6. Determination of Soluble Protein Content

Fresh leaf tissue (0.1 g) was frozen in liquid nitrogen and ground into a fine powder. The powder was immediately homogenized with 2 mL of 50 mM phosphate buffer (pH 7.0). After centrifugation at 8000 rpm for 10 min at 4 °C, the supernatant was collected as the soluble protein extract.

A 0.1 mL aliquot of the extract was mixed with 1 mL of Bradford reagent and incubated at room temperature for 5 min. Absorbance was measured at 595 nm using distilled water as the blank. A standard curve was constructed using bovine serum albumin (BSA; Beijing Solarbio Science & Technology Co., Ltd., Beijing, China) solutions ranging from 0 to 1 mg mL^−1^ [35]. Soluble protein content was calculated as follows:

Soluble protein content (mg g^−1^ FW) = C × V × DF/W,

where C is the soluble protein concentration determined from the standard curve, V is the total extraction volume, DF is the dilution factor, and W is the fresh weight of the sample.

#### 4.3.7. Determination of Ascorbate Peroxidase (APX) Activity

Fresh leaf tissue (0.2 g) was frozen in liquid nitrogen and ground into a fine powder. The powder was immediately homogenized with 2 mL of extraction buffer prepared by mixing equal volumes of 50 mM phosphate buffer (pH 7.0), 1 mM ascorbic acid, and 1 mM EDTA (Beijing Solarbio Science & Technology Co., Ltd., Beijing, China). The homogenate was centrifuged at 8700 rpm for 15 min at 4 °C, and the supernatant was collected as the enzyme extract and maintained at low temperature.

The reaction mixture contained 800 μL of 50 mM potassium phosphate buffer (pH 7.0), 100 μL of 0.5 mM ascorbic acid, and 50 μL of enzyme extract. The reaction was initiated by adding 0.5 mM H_2_O_2_. The decrease in absorbance at 290 nm was immediately recorded at 1 min intervals for 3–5 min [36]. For the blank, the enzyme extract was replaced with an equal volume of phosphate buffer. APX activity was calculated as follows:

APX activity (U g^−1^ FW) = (ΔA290 min^−1^ × Vt)/(ε × d × Ve × W),

where ΔA290 min^−1^ is the decrease in absorbance per minute, Vt is the total reaction volume in mL, ε is the molar extinction coefficient of ascorbate (2.8 mM^−1^ cm^−1^), d is the optical path length of the cuvette in cm, Ve is the volume of enzyme extract used in the reaction in mL, and W is the fresh weight of the sample in g.

#### 4.3.8. Determination of Catalase (CAT) Activity

Fresh leaf tissue (0.2 g) was frozen in liquid nitrogen and ground into a fine powder. The powder was immediately homogenized with 2 mL of 0.1 M phosphate buffer (pH 7.0). The homogenate was centrifuged at 8000 rpm for 10 min at 4 °C, and the supernatant was collected as the crude enzyme extract and maintained on ice.

The CAT reaction solution was prepared by mixing 5 mL of 0.1 M H_2_O_2_ with 20 mL of 0.1 M phosphate buffer (pH 7.0). A reaction mixture containing 10 μL of crude enzyme extract and 500 μL of CAT reaction solution was prepared. The decrease in absorbance at 240 nm was recorded at 1 min intervals for 3 min [37]. Distilled water was used as the blank.

CAT activity (U g^−1^ FW) = (ΔA240 min^−1^ × Vt)/(0.1 × Ve × W)

where one unit of CAT activity was defined as a decrease of 0.1 in absorbance at 240 nm per minute.

#### 4.3.9. Determination of Peroxidase (POD) Activity

Fresh leaf tissue (0.2 g) was frozen in liquid nitrogen and ground into a fine powder. The powder was immediately homogenized with 2 mL of 0.1 M phosphate buffer (pH 6.0). The homogenate was centrifuged at 8000 rpm for 10 min at 4 °C, and the supernatant was collected as the crude enzyme extract and maintained on ice.

The POD reaction solution was prepared by adding 28 μL of guaiacol to 50 mL of 0.1 M phosphate buffer (pH 6.0). The solution was stirred with gentle heating until the guaiacol was completely dissolved. After cooling, 19 μL of 30% H_2_O_2_ was added, and the solution was stored at 4 °C in the dark.

A reaction mixture containing 2 μL of crude enzyme extract and 300 μL of POD reaction solution was prepared. The increase in absorbance at 470 nm was recorded at 1 min intervals for 3 min [38]. A 0.1 M phosphate-buffered solution at pH 6.0 was used as the blank.

POD activity (U g^−1^ FW) = (ΔA470 min^−1^ × Vt)/(0.1 × Ve × W),

where one unit of POD activity was defined as an increase of 0.1 in absorbance at 470 nm per minute.

### 4.4. Transcriptome Sequencing and Differential Expression Analysis

Pretreatment control leaf samples and leaves collected after 1 and 5 days of exposure to 10 °C were used for transcriptome sequencing in ‘Musang King’ and ‘Monthong’. Three biological replicates were included for each cultivar and sampling stage, resulting in a total of 18 RNA-seq libraries: MKCK, MK1d, MK5d, MTCK, MT1d, and MT5d. Library construction and sequencing were performed by Beijing Tsingke Biotechnology Co., Ltd. (Beijing, China). Library quality was assessed using the Qsep-400 system (BiOptic Inc., New Taipei City, Taiwan), and paired-end sequencing was conducted on the DNBSEQ-T7 platform (MGI Tech Co., Ltd., Shenzhen, China) using combinatorial probe–anchor synthesis technology.

Raw reads were filtered by removing adapter-contaminated sequences, reads shorter than 100 bp after quality trimming, reads containing more than 10 ambiguous nucleotides, and reads in which more than 40% of bases had quality scores of Q ≤ 30. Clean reads were aligned to the durian reference genome using HISAT2 v2.2.1. Transcript reconstruction and gene-expression quantification were performed using StringTie v2.0.4, and expression levels were normalized as fragments per kilobase of transcript per million mapped reads (FPKM) and transcripts per million (TPM).

Differential-expression analysis was performed using DESeq2 v1.26.0. Genes with *p* < 0.05 and |log2FC| ≥ 1 were classified as differentially expressed according to the predefined criterion used throughout the candidate-prioritization workflow.

#### Musang King-Focused Discovery-Set Construction

To identify genes showing progressive transcriptional activation during chilling, genes significantly up-regulated at day 1 relative to the pretreatment control were intersected with genes showing further significant up-regulation from day 1 to day 5, using *p* < 0.05 and |log2FC| ≥ 1 for each comparison. This procedure was independently applied to ‘Musang King’ and ‘Monthong’. For construction of the Musang King-focused discovery set, genes that also fulfilled the same progressive-up-regulation criterion in ‘Monthong’ were not retained.

In parallel, genes showing significantly higher transcript abundance in ‘Musang King’ than in ‘Monthong’ at day 1 were intersected with those showing significantly higher abundance in ‘Musang King’ at day 5 using the same differential-expression threshold. The final discovery set was obtained by intersecting this cross-cultivar set with the Musang King-focused progressive-response set.

### 4.5. Identification and Classification of AP2/ERF Gene Family Members in Durian

The reference genome and annotation of durian were sourced from Figshare (https://figshare.com/articles/dataset/Durian_genome_annotation/25237591) (accessed on 8 April 2026) [39], while *Arabidopsis* genomic data were taken from the TAIR database (https://www.arabidopsis.org/) (accessed on 8 April 2026) [40]. Candidate AP2/ERF proteins were initially identified by BLASTP searches against the durian protein dataset using TBtools v2.326 [41]. In parallel, an HMM search was performed using the AP2-domain profile PF00847 obtained from Pfam. Candidates identified by the two approaches were merged, deduplicated, and subjected to domain confirmation using NCBI CDD and Pfam. The amino acid sequences of the AP2 domains were aligned using DNAMAN (version 6.0.3.99), and classified into subfamilies based on their structural features together with the amino acid composition at positions 14 and 19 of the AP2 domain. DREB members were ultimately renamed *DzDREB1* through *DzDREB81* in accordance with their physical distribution across the chromosomes for downstream analyses.

#### 4.5.1. Physicochemical Characterization and Subcellular Localization Prediction of the DREB Gene Family

The amino acid length, molecular mass, theoretical isoelectric point, instability index, aliphatic index, and grand average of hydropathicity of the DzDREB proteins were calculated using the Protein Parameter Calc function in TBtools [41]. Prediction of their subcellular distribution was performed via the WoLF PSORT platform (https://wolfpsort.hgc.jp/) (accessed on 9 April 2026) [42].

#### 4.5.2. Phylogenetic Tree Construction

A phylogenetic tree was constructed using MEGA12 with the maximum likelihood method and 1000 bootstrap replicates. The tree was visualized and annotated using iTOL (https://itol.embl.de/) (accessed on 9 April 2026) [43].

#### 4.5.3. Gene Structure, Conserved Domain, and Motif Analysis

The conserved domains were analyzed using NCBI CDD (https://www.ncbi.nlm.nih.gov/Structure/bwrpsb/bwrpsb.cgi) (accessed on 10 April 2026). The conserved motifs were identified using the MEME suite (https://meme-suite.org/meme/) (accessed on 10 April 2026), with the motif number set to 20. The gene structure was analyzed based on the genomic annotation. Visualization was accomplished using TBtools [41].

#### 4.5.4. Promoter cis-Regulatory Element Analysis

The 2000 bp sequences upstream of each gene were extracted as putative promoter regions. Cis-regulatory elements were analyzed using PlantCARE (https://bioinformatics.psb.ugent.be/webtools/plantcare/html/) (accessed on 10 April 2026), and the results were visualized using TBtools [41].

#### 4.5.5. Synteny Analysis

Based on the genome annotations of durian and *Arabidopsis*, intra-species and inter-species synteny analyses were performed using TBtools [41] and MCScanX (default parameters). The synteny relationships were visualized using the Advanced Circos function in TBtools v2.326.

#### 4.5.6. GO and KEGG Enrichment Analysis

Gene annotations were obtained using eggNOG-mapper (http://eggnog-mapper.embl.de/) (accessed on 15 April 2026) [44]. Enrichment analysis and bar chart visualization were performed using TBtools [41].

### 4.6. RNA Extraction, qRT-PCR, and Seasonal Field-Expression Profiling

Total RNA was isolated via the CTAB protocol and stored at −80 °C until use. RNA concentration and purity were determined using an ND-100C microvolume spectrophotometer (Hangzhou Miu Instruments Co., Ltd., Hangzhou, China). Gene-specific qRT-PCR primers (listed in Supplementary Table S4) were designed using the NCBI Primer-BLAST web interface (https://www.ncbi.nlm.nih.gov/tools/primer-blast/) (accessed on 25 May 2026), with *Dz4GAPDH09.1422* serving as the reference gene. All oligonucleotide primers were custom-synthesized by Beijing Tsingke Biotechnology Co., Ltd. Following concentration normalization, first-strand cDNA was reverse-transcribed from equal amounts of RNA using the SPARKscript II RT Plus Kit with gDNA cleaner (SparkJade) (Shandong Sikejie Biotechnology Co., Ltd., Qingdao, China). Real-time PCR amplifications were run on an ABI 7500 system (Life Technologies, Carlsbad, CA, USA), with each 20 μL reaction containing 10 μL of 2×SYBR qPCR Mix, 1 μL of cDNA template, 0.4 μL each of forward and reverse primers, 0.4 μL of ROX Reference Dye II, and 7.8 μL of nuclease-free water. Both reverse transcription and qPCR reagents were sourced from Shandong Sikejie Biotechnology Co., Ltd. (Qingdao, China). The relative transcript abundances were calculated using the 2^−ΔΔCt^ method. The field qRT-PCR data were used to characterize seasonal expression patterns of the prioritized DzDREB genes.

### 4.7. Statistical Analysis

Physiological data are presented as the mean ± standard deviation of three biological replicates. Differences among treatment durations within each cultivar were evaluated using one-way analysis of variance followed by Tukey’s multiple-comparison test. These analyses were intended to characterize temporal changes within each cultivar; no formal cultivar × treatment-duration interaction was tested. Differences were considered statistically significant at *p* < 0.05.

Physiological data were analyzed using IBM SPSS Statistics version 27.0.1.0 and plotted using OriginPro 2024 version 10.1.0.178. qRT-PCR data were analyzed using ΔCt values, and the figures were generated using GraphPad Prism version 10.1.2.

## 5. Conclusions

This study characterized phenotypic, physiological, and transcriptional responses of durian seedlings during prolonged chilling and performed a genome-wide analysis of the DzDREB family. Consistent with previous reports placing ‘Musang King’ above ‘Monthong’ in chilling tolerance, ‘Monthong’ showed earlier and visually more extensive leaf abscission, while the two cultivars exhibited contrasting temporal patterns of MDA accumulation and antioxidant enzyme activities. A hypothesis-driven Musang King-focused transcriptomic strategy generated a 331-gene discovery set characterized by progressive induction and preferential transcript abundance in ‘Musang King’. Subsequent genome-wide analysis identified 81 DzDREB genes classified into six subgroups. Integration of family-wide transcriptomic response patterns, stress-related functional annotations, and promoter cis-regulatory elements supported the prioritization of a ten-gene DzDREB panel. Exploratory seasonal field-expression profiling provided additional supporting evidence for seven of the ten prioritized candidates, among which *DzDREB36*, *DzDREB45*, *DzDREB62*, and *DzDREB66* showed comparatively stronger cross-dataset expression support. These genes represent priority candidates for future functional studies aimed at elucidating the molecular basis of differential chilling responses in durian.

## Data Availability

The original contributions presented in this study are included in the article/Supplementary Materials. Further inquiries can be directed to the corresponding author.

## Supplementary Materials

The following supporting information can be downloaded at https://www.mdpi.com/article/10.3390/ijms27188065/s1.
