# Supplementary material for: Physiological Responses to Chilling Stress and Transcriptome-Assisted Genome-Wide Characterization of the DREB Gene Family in Durian
Source: Int J Mol Sci. 2026 Sep 10;27(18):8065. doi: 10.3390/ijms27188065 (PMC13607584; doi:10.3390/ijms27188065)
Supplement: Supplementary file 1 [file ijms-27-08065-s001.zip › Supplementary Table S1.pdf]

Supplementary Table S1 Quality control of raw sequencing data for durian

Sample	Raw Reads	Raw Bases	Clean Reads	Clean Bases	Effective Rate(%)	GC(%)	Q20(%)	Q30(%)
MK1d_1	46,132,286	6,919,842,900	41,273,832	6,174,891,774	89.23	43.04	97.70	92.71
MK1d_2	41,628,662	6,244,299,300	37,372,756	5,583,939,831	89.42	42.74	97.78	93.19
MK1d_3	46,577,112	6,986,566,800	41,710,250	6,228,229,204	89.15	42.68	97.73	93.03
MK5d_1	50,369,458	7,555,418,700	44,349,482	6,636,551,996	87.84	44.06	97.50	92.04
MK5d_2	43,849,312	6,577,396,800	38,566,896	5,769,823,661	87.72	43.94	97.51	92.14
MK5d_3	42,775,698	6,416,354,700	37,829,918	5,660,619,708	88.22	43.94	97.53	92.22
MKCK_1	53,973,028	8,095,954,200	49,538,088	7,420,479,057	91.66	42.73	98.53	94.55
MKCK_2	39,680,060	5,952,009,000	35,544,252	5,323,559,981	89.44	42.91	98.33	93.88
MKCK_3	38,860,056	5,829,008,400	35,413,948	5,304,592,732	91.00	42.95	98.47	94.39
MT1d_1	39,942,818	5,991,422,700	35,325,924	5,283,871,858	88.19	42.63	97.49	92.02
MT1d_2	44,573,198	6,685,979,700	39,483,842	5,903,910,645	88.30	42.76	97.61	92.35
MT1d_3	45,513,682	6,827,052,300	40,549,646	6,055,085,174	88.69	42.57	97.65	92.78
MT5d_1	47,317,910	7,097,686,500	41,770,284	6,246,627,775	88.01	43.14	97.54	92.17
MT5d_2	46,071,682	6,910,752,300	41,250,370	6,161,545,000	89.16	42.82	97.68	92.84
MT5d_3	48,440,698	7,266,104,700	43,703,288	6,488,759,795	89.30	42.87	97.99	93.85
MTCK_1	46,758,482	7,013,772,300	42,059,282	6,283,556,833	89.59	43.2	97.73	93.13
MTCK_2	49,164,488	7,374,673,200	43,781,056	6,537,526,090	88.65	42.82	97.66	92.81
MTCK_3	45,684,862	6,852,729,300	40,861,674	6,103,105,455	89.06	42.42	97.67	92.81
