# Supplementary material for: Physiological Responses to Chilling Stress and Transcriptome-Assisted Genome-Wide Characterization of the DREB Gene Family in Durian
Source: Int J Mol Sci. 2026 Sep 10;27(18):8065. doi: 10.3390/ijms27188065 (PMC13607584; doi:10.3390/ijms27188065)
Supplement: Supplementary file 1 [file ijms-27-08065-s001.zip › Supplementary Table S2.pdf]

Supplementary Table S2 Comparative classification of AP2/ERF gene family in durian and Arabidopsis

	AP2	RAV	ERF	DREB	Solosist	Total
Durian	35	8	123	81	0	247
Arabidopsis thaliana	18	6	65	57	1	147
