# Supplementary material for: Physiological Responses to Chilling Stress and Transcriptome-Assisted Genome-Wide Characterization of the DREB Gene Family in Durian
Source: Int J Mol Sci. 2026 Sep 10;27(18):8065. doi: 10.3390/ijms27188065 (PMC13607584; doi:10.3390/ijms27188065)
Supplement: Supplementary file 1 [file ijms-27-08065-s001.zip › Supplementary Table S3.pdf]

Supplementary Table S3 Features of DzDREB gene family

Gene name	Sequence ID	family	Amino acid length	Molecular Weight	Theoretical pI	Instability Index	Aliphatic Index	Grand Average of Hydropathicity	Subcellular Localization
DzDREB1	*evm.model.GRLG01.119*	A-2	296	33576.97	8.60	53.20	64.66	-0.71	nucleus
DzDREB2	*evm.model.GRLG01.427*	A-4	691	78999.70	6.08	46.77	82.85	-0.26	nucleus
DzDREB3	*evm.model.GRLG01.429*	A-4	209	21949.53	5.93	44.49	71.24	-0.33	chloroplast
DzDREB4	*evm.model.GRLG01.932*	A-2	459	52289.89	5.04	42.96	61.81	-0.86	nucleus
DzDREB5	*evm.model.GRLG01.1284*	A-5	229	25144.38	5.05	65.93	69.04	-0.36	nucleus
DzDREB6	*evm.model.GRLG01.1374*	A-6	358	39788.33	6.72	59.81	58.35	-0.71	nucleus
DzDREB7	*evm.model.GRLG01.1661*	A-5	151	16992.05	9.54	52.50	60.79	-0.89	nucleus
DzDREB8	*evm.model.GRLG01.1877*	A-2	214	23243.94	4.88	46.93	58.46	-0.57	nucleus
DzDREB9	*evm.model.GRLG02.1058*	A-6	758	84008.73	6.44	56.18	72.12	-0.50	nucleus
DzDREB10	*evm.model.GRLG03.766*	A-6	506	56265.34	5.38	61.19	56.34	-0.81	nucleus
DzDREB11	*evm.model.GRLG04.735*	A-6	380	41907.07	8.89	55.69	74.71	-0.56	nucleus
DzDREB12	*evm.model.GRLG04.1278*	A-4	248	27580.58	6.76	50.05	64.64	-0.71	nucleus
DzDREB13	*evm.model.GRLG04.1350*	A-4	209	23256.03	9.23	49.20	58.85	-0.78	nucleus
DzDREB14	*evm.model.GRLG04.1415*	A-4	179	19946.68	6.53	44.20	78.60	-0.38	nucleus
DzDREB15	*evm.model.GRLG05.66*	A-4	254	27604.38	4.92	48.23	59.17	-0.59	nucleus
DzDREB16	*evm.model.GRLG05.327*	A-5	230	25425.71	5.02	55.29	56.83	-0.76	nucleus
DzDREB17	*evm.model.GRLG05.486*	A-4	257	28043.33	5.20	67.27	66.38	-0.52	nucleus
DzDREB18	*evm.model.GRLG05.2230*	A-1	581	65072.26	6.35	49.71	89.00	-0.24	nucleus
DzDREB19	*evm.model.GRLG06.577*	A-4	274	29989.17	5.13	58.68	71.28	-0.48	nucleus
DzDREB20	*evm.model.GRLG07.156*	A-4	258	28655.21	6.62	45.41	62.79	-0.64	nucleus
DzDREB21	*evm.model.GRLG07.258*	A-4	173	18762.87	5.44	44.59	63.93	-0.63	nucleus
DzDREB22	*evm.model.GRLG07.374*	A-6	288	32288.55	9.04	46.26	69.83	-0.58	nucleus
DzDREB23	*evm.model.GRLG07.677*	A-1	223	24492.06	5.38	47.67	47.44	-0.71	nucleus
DzDREB24	*evm.model.GRLG08.279*	A-4	285	31756.66	7.72	49.84	63.09	-0.58	nucleus
DzDREB25	*evm.model.GRLG08.431*	A-1	231	26017.14	5.73	59.37	68.92	-0.63	nucleus

DzDREB26	*evm.model.GRLG08.432*	A-4	265	28569.13	5.75	52.00	68.98	-0.43	nucleus
DzDREB27	*evm.model.GRLG08.1096*	A-1	264	29235.67	5.63	56.78	65.45	-0.62	nucleus
DzDREB28	*evm.model.GRLG08.1097*	A-4	251	26757.97	6.32	64.03	58.80	-0.71	nucleus
DzDREB29	*evm.model.GRLG08.1822*	A-4	247	28075.46	9.22	64.23	51.86	-0.81	nucleus
DzDREB30	*evm.model.GRLG09.151*	A-6	382	42697.11	5.81	65.03	54.40	-0.74	nucleus
DzDREB31	*evm.model.GRLG09.188*	A-4	197	22313.14	7.71	65.84	56.04	-0.72	nucleus
DzDREB32	*evm.model.GRLG09.447*	A-5	232	25347.50	9.07	36.31	75.26	-0.51	nucleus
DzDREB33	*evm.model.GRLG10.314*	A-4	221	24256.27	5.82	69.25	69.82	-0.52	nucleus
DzDREB34	*evm.model.GRLG10.497*	A-2	299	33645.99	6.34	53.88	75.08	-0.60	nucleus
DzDREB35	*evm.model.GRLG10.773*	A-2	264	29043.34	5.11	42.28	55.49	-0.71	nucleus
DzDREB36	*evm.model.GRLG12.27*	A-2	526	59556.96	5.20	42.68	56.56	-0.91	nucleus
DzDREB37	*evm.model.GRLG12.339*	A-4	232	24764.95	6.32	47.45	77.16	-0.21	nucleus
DzDREB38	*evm.model.GRLG12.341*	A-4	242	26670.81	6.77	78.55	59.75	-0.66	nucleus
DzDREB39	*evm.model.GRLG12.541*	A-2	302	34287.55	5.65	58.00	66.66	-0.68	chloroplast
DzDREB40	*evm.model.GRLG12.669*	A-2	272	29908.28	5.41	37.28	48.90	-0.69	nucleus
DzDREB41	*evm.model.GRLG12.892*	A-3	330	36124.35	9.76	55.71	62.67	-0.57	nucleus
DzDREB42	*evm.model.GRLG12.1084*	A-2	1055	117139.30	5.81	51.34	70.35	-0.55	nucleus
DzDREB43	*evm.model.GRLG13.344*	A-4	251	27304.94	4.85	41.45	56.41	-0.67	nucleus
DzDREB44	*evm.model.GRLG13.514*	A-5	173	19633.03	8.94	48.52	54.74	-0.81	cytoplasm
DzDREB45	*evm.model.GRLG13.555*	A-5	227	25309.49	4.76	47.53	54.19	-0.85	cytoplasm
DzDREB46	*evm.model.GRLG13.650*	A-4	272	29777.23	5.13	56.15	69.19	-0.51	nucleus
DzDREB47	*evm.model.GRLG14.409*	A-6	462	51697.75	5.97	63.03	60.02	-0.73	nucleus
DzDREB48	*evm.model.GRLG15.195*	A-4	993	112031.50	7.00	47.19	80.77	-0.37	nucleus
DzDREB49	*evm.model.GRLG15.291*	A-1	639	70991.19	7.19	39.46	90.38	0.03	plasma membrane
DzDREB50	*evm.model.GRLG15.412*	A-6	323	36350.06	9.10	44.37	72.54	-0.63	nucleus
DzDREB51	*evm.model.GRLG15.744*	A-1	236	25817.86	5.99	56.09	50.55	-0.67	nucleus
DzDREB52	*evm.model.GRLG15.745*	A-4	289	31047.82	5.85	57.93	59.79	-0.58	nucleus
DzDREB53	*evm.model.GRLG15.1319*	A-4	259	29273.18	5.99	62.52	75.02	-0.47	nucleus
DzDREB54	*evm.model.GRLG16.38*	A-4	173	18901.20	5.80	52.83	58.79	-0.58	nucleus

DzDREB55	*evm.model.GRLG16.45*	A-5	162	17547.89	8.66	65.10	75.37	-0.24	nucleus
DzDREB56	*evm.model.GRLG16.403*	A-5	642	71291.17	5.77	53.22	77.06	-0.25	chloroplast
DzDREB57	*evm.model.GRLG16.456*	A-6	361	40425.08	6.08	68.63	57.29	-0.72	nucleus
DzDREB58	*evm.model.GRLG16.694*	A-5	493	56556.82	6.05	50.02	77.08	-0.68	nucleus
DzDREB59	*evm.model.GRLG16.852*	A-2	230	25167.90	4.97	46.58	58.22	-0.61	mitochondrion
DzDREB60	*evm.model.GRLG17.143*	A-4	298	33097.69	6.67	52.42	62.28	-0.70	nucleus
DzDREB61	*evm.model.GRLG19.69*	A-3	288	31142.36	9.38	52.44	59.62	-0.70	nucleus
DzDREB62	*evm.model.GRLG19.474*	A-2	258	28251.66	5.05	44.64	65.50	-0.58	nucleus
DzDREB63	*evm.model.GRLG19.802*	A-2	377	40860.02	4.40	43.24	78.12	-0.62	nucleus
DzDREB64	*evm.model.GRLG20.421*	A-4	251	27495.41	5.49	57.18	59.08	-0.67	nucleus
DzDREB65	*evm.model.GRLG20.549*	A-5	149	16209.78	6.91	39.22	50.00	-0.84	nucleus
DzDREB66	*evm.model.GRLG20.575*	A-5	228	25156.31	4.45	52.31	51.40	-0.80	nucleus
DzDREB67	*evm.model.GRLG20.680*	A-4	261	28528.75	5.31	71.06	60.92	-0.53	nucleus
DzDREB68	*evm.model.GRLG20.1412*	A-6	365	40669.34	5.65	60.57	64.74	-0.63	nucleus
DzDREB69	*evm.model.GRLG20.1605*	A-5	180	20506.77	9.39	33.53	60.72	-1.03	nucleus
DzDREB70	*evm.model.GRLG21.436*	A-6	373	41056.14	8.88	42.39	70.11	-0.60	nucleus
DzDREB71	*evm.model.GRLG22.707*	A-6	366	40709.90	5.64	58.86	57.90	-0.69	nucleus
DzDREB72	*evm.model.GRLG22.915*	A-5	175	19501.69	9.45	38.77	64.74	-0.83	nucleus
DzDREB73	*evm.model.GRLG24.1088*	A-4	157	17493.75	8.30	52.48	63.50	-0.56	chloroplast
DzDREB74	*evm.model.GRLG24.1343*	A-4	327	36360.26	6.11	60.45	65.14	-0.74	nucleus
DzDREB75	*evm.model.GRLG25.1094*	A-4	244	26683.23	5.29	70.19	62.83	-0.59	nucleus
DzDREB76	*evm.model.GRLG26.769*	A-6	418	46050.21	6.12	49.27	63.04	-0.64	nucleus
DzDREB77	*evm.model.GRLG27.107*	A-4	193	21408.36	5.19	43.73	83.99	-0.27	nucleus
DzDREB78	*evm.model.GRLG27.108*	A-4	212	23301.29	7.75	50.69	64.10	-0.55	nucleus
DzDREB79	*evm.model.GRLG27.117*	A-5	173	19167.56	7.87	63.01	63.76	-0.55	nucleus
DzDREB80	*evm.model.GRLG27.600*	A-2	173	19429.80	5.31	47.29	68.79	-0.60	nucleus
DzDREB81	*evm.model.GRLG28.648*	A-4	281	31040.43	4.92	54.32	67.40	-0.45	nucleus
