# Supplementary material for: Physiological Responses to Chilling Stress and Transcriptome-Assisted Genome-Wide Characterization of the DREB Gene Family in Durian
Source: Int J Mol Sci. 2026 Sep 10;27(18):8065. doi: 10.3390/ijms27188065 (PMC13607584; doi:10.3390/ijms27188065)
Supplement: Supplementary file 1 [file ijms-27-08065-s001.zip › Supplementary Table S4.pdf]

Table S4 Sequences of primers for quantitative real-time PCR

Gene Name	Sequence ID	Forward primer sequence (5'–3')	Reverse primer sequence (5'–3')
DzDREB6	*evm.model.GRLG01.1374*	GGCAACGTCACTGGGGAA	GCCAAGGCTGCTTCCTCA
DzDREB7	*evm.model.GRLG01.1661*	ACTTGCGTGGACCTTCCG	TCGACTTTGGCCCCAACC
DzDREB25	*evm.model.GRLG08.431*	TGCCTGTGCCAGCTTCAA	ACTTTCCTGCCTCGCGTC
DzDREB36	*evm.model.GRLG12.27*	GCGAAGACGATGGCCTCA	AGTTCTCCGGGCCTCCTT
DzDREB42	*evm.model.GRLG12.1084*	TGGGGTAAGTGGGTGGCT	GCGGCCTGGAAAGCAGTA
DzDREB45	*evm.model.GRLG13.555*	AAGCCAACCTGCGGACAA	TACGCTCACGGCTGTTGG
DzDREB48	*evm.model.GRLG15.195*	AGAGAGGAGCAAGGCGGA	ACCGGATCACTTGCGACG
DzDREB57	*evm.model.GRLG16.456*	GGCAACGTCACTGGGGAA	GCCAAGGCTGCTTCCTCA
DzDREB62	*evm.model.GRLG19.474*	AGGGCTCGAAGAAGGGGT	CCGTTAGGCGCTCGGATT
DzDREB66	*evm.model.GRLG20.575*	GGTCTTACGACTCGGCGG	AGCGACCTCCCTCCAACT
DzICE5	*evm.model.GRLG01.1666*	CAATGCAGCTGCCTCCCA	AAATGCGCCTGCTCCTGT
DzICE8	*evm.model.GRLG02.1833*	GGATGGAGTTCCGAGCCC	AAACGTTGACCCTGGGCC
DzICE22	*evm.model.GRLG08.148*	CCAAGTCCTCCTTCGCCG	TCGATGAGGGCGTGGAGA
DzICE33	*evm.model.GRLG09.1521*	GTTTTGTGCTCGCAGGCC	CATCCTGCCCTTCCCTGC
DzICE34	*evm.model.GRLG10.1251*	GTTGTGCTGTGAGGACCGA	AGCGTCGCCATTTCTGCT
DzICE57	*evm.model.GRLG17.499*	TCGGCAGAGAGACACCGT	GCTTTGTCCATGCGCGAC
DzICE71	*evm.model.GRLG20.1612*	TGTCACGGTCACTGCCAC	CCCTCCACTCCCTCTCCC
DzICE77	*evm.model.GRLG22.922*	ATCACGGTCAGCGACACC	CCTTCACCCCCTCTCCCA
DzICE79	*evm.model.GRLG24.690*	GGATGCCCATCCGGTCTC	CCTGGGGCTCGAATGGTG
DzICE82	*evm.model.GRLG24.1089*	GGGTCCTTGGTTGGGGTG	CGTCTTCGAGCCGCTGAT
Dz4GAPDH09.1422	*evm.model.GRLG09.1422*	TGGGCTGAGACAGGAGCT	CCACAACAAACATGGGGGC
